# Lessons from the Nationwide Surveillance of SARS-CoV-2 Surges in Japan

**DOI:** 10.31662/jmaj.2021-0091

**Published:** 2021-07-06

**Authors:** Masafumi Seki

**Affiliations:** 1 Division of Infectious Diseases and Infection Control, Faculty of Medicine, Tohoku Medical and Pharmaceutical University, Sendai, Japan

**Keywords:** COVID-19, Infectivity, lethality, Molecular Epidemiology, influenza, Human Immunodeficiency virus

Coronavirus infectious disease 2019 (COVID-19), which continues to spread globally, has become the primary focus of infectious disease research since 2020 ^(1)^. Humans have experienced acute epidemic viral infections, with influenza being typical, but a pandemic of this size has not been seen since the “Spanish flu” of 1918 ^(2)^. Severe acute respiratory syndrome coronavirus 2 (SARS-CoV-2) appears similar to the other respiratory viruses, including influenza; however, critical differences have been found between COVID-19 and influenza.

Compared with these previous illnesses, COVID-19 has somewhat stronger infectivity but lower lethality. Although COVID-19 was initially seen as a viral infection similar to seasonal influenza, it soon became empirically clear that it is much more serious ^(1)^.

Although the infectivity of COVID-19 seems to be about the same as that of influenza, in situations involving crowded places, close-contact settings and confined and enclosed spaces―known as the “three Cs”―a “cluster” is generated wherein nearly all the people present become infected at a rate comparable with that of the measles ^(3), (4)^. This situation may be affected by a subtle mechanism resembling the human immunodeficiency viruses wherein the virus is able to propagate while evading attack by the human immune system. As a result, infected persons can remain asymptomatic for about a week after being infected.

Although the overall case fatality rate in Japan remains quite low, it is still much higher than that for influenza, and the lethality of the disease has been especially severe in the aged; therefore, we must remain vigilant ^(1)^. Although most young people have experienced relatively mild clinical manifestations of COVID-19, pneumonia presenting with characteristic bilateral ground-glass opacity chest has been observed on computed tomography scans, even in patients who show no or only mild symptoms. In contrast, serious and potentially fatal clinical manifestations of COVID-19 have been seen at a very high rate among the elderly, especially those with preexisting conditions ^(1)^. Hence, age remains one of the most important factors to determine the prognosis of patients infected with SARS-CoV-2. Therefore, COVID-19 can be considered a formidable infectious disease with two distinct manifestations.

These two serious characteristics of SARS-CoV-2―infectivity and lethality―have been confirmed epidemiologically through an analysis of nationwide surveillance data during the first to the third wave of COVID-19 in Japan ^(5)^.

The authors of that study demonstrated that the first wave in spring 2020 was characterized by the detection of infected patients from China followed by a notification of sporadic cases, including the “Diamond Princess” cruise ship and music clubs in Osaka ^(3), (4)^. Seemingly healthy people could spread SARS-CoV-2 during intensive activities in enclosed environments. It has been clear that infected asymptomatic individuals can transmit the virus as soon as 2 days after infection ^(5)^.

The second wave in summer 2020 showed a large increase in notifications and a younger age distribution. Testing, especially polymerase chain reaction tests, became more available in the summer compared with that in the spring, and the test positivity declined. Moreover, it was found that the younger population was experiencing substantially milder cases of the disease. Although the mortality associated with COVID-19 remained low, severe and fatal cases skewed toward the aged, particularly older men. The data suggested that the mortality remained lower even when including the number of deaths at 1 month after the end of each wave to account for any “time lag ^(5)^.”

The third wave in winter 2020 was characterized by steady notifications and a relatively high prevalence of hospitalized cases, and resulted in substantially higher mortality and morbidity, surpassing both the total and fatal case counts from the first two waves combined. Although the magnitude of the increases in mortality and morbidity varied, a surge was observed in urban and rural areas nationwide. To stimulate the economy, “Go Out To Eat” and “Go Travel” campaigns were introduced by the Japanese government to encourage dining out and taking domestic trips, respectively, which might have affected and exacerbated the COVID-19 pandemic ^(5)^.

In all three COVOD-19 waves in Japan, the characteristic infectivity and lethality of SARS-CoV-2 was observed, but the propagation of the disease and its mortality varied depending on the situation and the backgrounds of the patients. The importance of molecular epidemiology has become increasingly apparent. If we continue to carry out appropriate nationwide surveillance, we should be able to predict and prepare for the next wave. Although we are already in the midst of a fourth wave, which is larger than the previous three, and numerous mutations of the virus with varying degrees of infectivity and lethality have already been observed, we can overcome this crisis by the rapid and widespread distribution of novel COVID-19 vaccines and continued interventions such as universal masking and hand washing.

## Article Information

### Conflicts of Interest

None
